# Comparative thermodynamic analysis in solution of a next generation antibody mimetic to VEGF

**DOI:** 10.1039/c8ra07059h

**Published:** 2018-10-19

**Authors:** Hanieh Khalili, Steve Brocchini, Peng Tee Khaw, Sergey K. Filippov

**Affiliations:** UEL School of Health, Sport and Bioscience London UK h.khalili@uel.ac.uk hanieh.khalili@ucl.ac.uk; UCL School of Pharmacy London UK; National Institute for Health Research (NIHR) Biomedical Research Centre at Moorfields Eye Hospital NHS Foundation Trust, UCL Institute of Ophthalmology London UK; Institute of Macromolecular Chemistry Prague Czech Republic sfill225@gmail.com

## Abstract

An antibody mimetic known as Fab–PEG–Fab (FpF) is a stable bivalent molecule that may have some potential therapeutic advantages over IgG antibodies due to differences in their binding kinetics as determined by surface plasmon resonance. Here we describe the thermodynamic binding properties to vascular endothelial growth factor (VEGF) of the FpF antibody mimetics derived from bevacizumab and ranibizumab. Bevacizumab is an IgG antibody and ranibizumab is an antibody fragment (Fab). Both are used clinically to target VEGF to inhibit angiogenesis. FpF_beva_ displayed comparable binding affinity (KD) and binding thermodynamics (Δ*H* = −25.7 kcal mole^−1^ and Δ*S* = 14 kcal mole^−1^) to bevacizumab (Δ*H* = −25 kcal mole^−1^, Δ*S* = 13.3 kcal mole^−1^). FpF_rani_ interactions with VEGF were characterised by large favourable enthalpy (Δ*H* = −42 kcal mole^−1^) and unfavourable entropy (Δ*S* = 31 kcal mole^−1^) changes compared to ranibizumab (Δ*H* = −18.5 kcal mole^−1^ and Δ*S* = 6.7 kcal mole^−1^), which being a Fab, is mono-valent. A large negative entropy change resulting in binding of bivalent FpF to homodimer VEGF might be due to the conformational change of the flexible regions of the FpF upon ligand binding. Mono-valent Fab (*i.e.* ranibizumab or the Fab derived from bevacizumab) displayed a larger degree of freedom (smaller unfavourable entropy) upon binding to homodimer VEGF. Our report describes the first comprehensive enthalpy and entropy compensation analysis for FpF antibody mimetics. While the FpFs displayed similar thermodynamics and binding affinity to the full IgG (*i.e.* bevacizumab), their enhanced protein stability, slower dissociation rate and lack of Fc effector functions could make FpF a potential next-generation therapy for local tissue-targeted indications.

## Abstract 2

ITC illustrated similar binding thermodynamics for anti-VEGF IgG and FpFs. Bivalent FpF_rani_ displayed larger enthalpy and entropy than monovalent ranibizumab.

## Graphical abstract

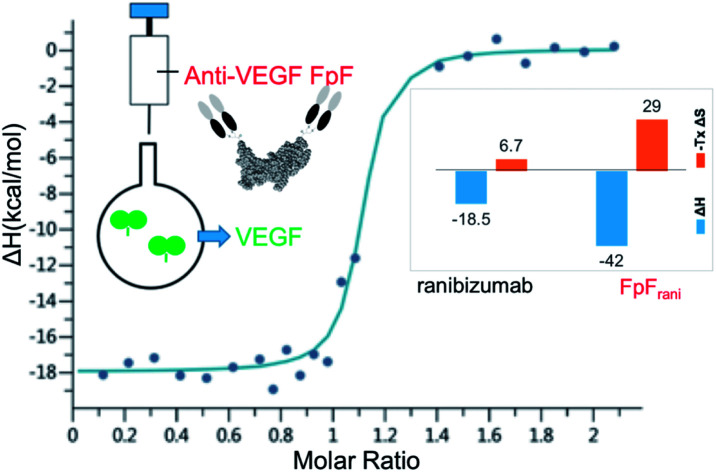

## Introduction

**Fig. 1:**
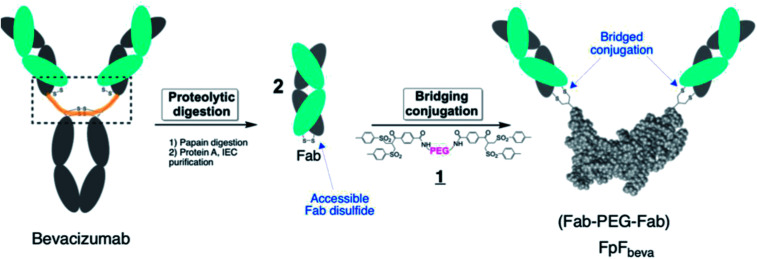
Synthetic route to make FpF_beva_ from bevacizumab. Fab_beva_ was obtained by papain enzymatic digestion of bevacizumab and purified using protein A and ion-exchange chromatography (IEC). The accessible interchange disulfide in the Fab_beva_ was then reduced by DTT and conjugated with PEG-di(bis-sulfone) 1. Each bis-sulfone moiety in reagent 1 undergoes site-specific conjugation with the two cysteine thiols from a disulfide bond by a sequence of addition–elimination reactions to insert a stable 3-carbon methylene bridge between the two thiols of the original disulfide bond.

IgG antibodies are widely used medicines that can have high affinity for a specific biological epitope. IgGs are bivalent with two Fabs that are each able to bind to the target epitope. High affinity is achieved by (i) the inherent binding of the complementary determinant region (CDR) in each Fab and (ii) the cooperative binding that is possible because there are two Fabs in an IgG antibody (*i.e.* avidity).^1^ The IgG is bivalent due to the presence of the 2 Fabs, which are essentially linked together through the antibody hinge region as if each Fab is bound at the end of linear molecule (Fig. 1). The cooperative binding of the two Fabs in an IgG is achieved by the flexibility provided in the hinge.^1,2^

Using a site-selective conjugation strategy to chemically modify Fabs in the region where they are naturally bound to the hinge provided the means to make Fab–PEG–Fab (FpF) molecules that are IgG mimetics.^3^ FpFs derived from IgG Fabs have been found to have enhanced stability with comparable affinity to the parent IgG. Improved stability is important to formulate more concentrated solutions to decrease the frequency of dose administration.^4,24^ Interestingly FpFs tend to display a slower dissociation rate as determined by surface plasmon resonance (SPR) studies compared to IgG molecules, which could have important therapeutic advantages.^3^

FpFs are prepared from the PEG-di(bis-sulfone) 1 which undergoes site-specific disulfide rebridging conjugation with the accessible Fab disulfide (Fig. 1). The FpFs are derived from bevacizumab and from ranibizumab which target anti-vascular endothelial growth factor (VEGF). Ranibizumab is a Fab and is clinically approved to treat wet age related macular degeneration (AMD) which is one of the main causes of blindness in the elderly. AMD is characterised by the over expression of VEGF which causes angiogenesis in the back of the eye resulting in loss of retinal function.^27^ Bevacizumab is an IgG that is targeted to VEGF, but is labelled for systemic parenteral use to treat cancer. Bevacizumab is clinically used off label to treat AMD. Inhibition and neutralization of VEGF is the most effective way to treat wet AMD and has revolutionised the treatment of neovascular and vascular permeability disorders of the retina.^5^

One advantage of the FpF as a IgG antibody mimetic is that there is no fragment crystallizable region (Fc). The Fc region in full IgG antibodies can act to cause effector functions and recycling, which are not required in some applications (*e.g.* opthalmic). IgGs are taken up by the retinal pigment epithelium cells (RPE) through binding of Fc to Fc-receptor (FcRn) to accelerate IgG elimination from the back of the eye.^6–9^ Inducing effector functions precludes the use of antibodies to treat acute inflammatory conditions in ocular tissue. Often the causes of serious inflammation are not known, so using a medicine that might cause additional inflammation must be avoided. Another important attribute of FpF antibody mimetics is the replacement of the hinge region with the flexible PEG and rebridged disulfides in the FpF molecule which are more stable than the hinge disulfide and single chain peptide chains that make up the IgG hinge region. The hinge region in a IgG is susceptible to degradation and disulfide scrambling. Molecules that are designed from long duration of action (*e.g.* 2 months) must be physicochemically stable.

Isothermal titration calorimetry (ITC) is used extensively to study ligand–macromolecule interactions in solution.^10^ Protein–protein studies dominate, but many types of interactions have been examined, *e.g.* protein–DNA, protein–lipid and protein–carbohydrate.^11^ ITC is an effective method to quantify the thermodynamic changes in solution that are associated with the binding interactions specific to antibody–antigen complexation, which typically progresses *via* an enthalpy driven process.

We have previously shown^3^ that FpFs displayed similar binding affinity (KD) to a full IgG using enzyme-linked immunosorbent assay (ELISA) and surface plasmon resonance (SPR) analysis. However, the KD derived from ELISA and SPR methods require either the ligand or the antibody to be immobilized which limits the ability to obtain a thermodynamic profile of the binding interactions.^25^ ITC allows the study of both the antibody and ligand in solution which is where the *in vivo* interactions of a circulating ligand with an antibody occur.^11,25^ In this paper we determine the thermodynamic parameters of an anti-VEGF FpF using ITC. These experiments reveal fundamental information on the mechanism of interaction and stoichiometry of binding^12^ between the FpF and its soluble ligand. It is not possible to determine the thermodynamic parameters using SPR and ELISA techniques. Since the FpFs are derived from a much larger molecular weight, non-peptide linking molecule (PEG) compared to how the Fabs are linked together in an IgG (hinge), we wanted to understand what the entropic costs to FpF binding would be.

Binding interactions can involve an enthalpy driven process which involves formation of favourable interactions at the molecular recognition interface and an entropy driven process which involves the release of surface bound solvent molecules at the molecular recognition interface.^12,13^ In some cases, a binding process can be both enthalpy and entropy driven. We aimed to use ITC to disentangle these thermodynamic processes to better understand the binding interactions between an IgG and FpF in solution. Our data indicate that the IgG hinge region and the FpF linker do not change the thermodynamic profile for binding to VEGF. This is quite remarkable considering the structural differences between IgGs and FpFs. More interestingly, when the FpF was synthesised from ranibizumab (a Fab), a larger unfavourable entropy resulted in FpF_rani_ compared to ranibizumab suggesting a tighter binding for FpF to VEGF.

## Results

### Binding thermodynamics of antibody mimetic to VEGF is enthalpy driven

**Fig. 2:**
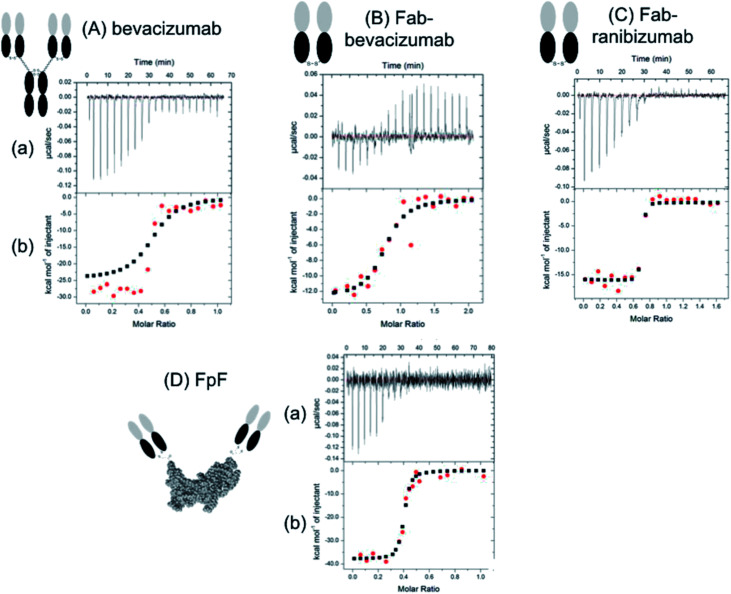
Representative data from an lTC experiment. Panel (a) shows the raw heat data obtained over a series of injections. Panel (b) shows a binding isotherm created by plotting the areas under the peaks in panel against the molar ratio of ligand added to macromolecule present in the cell.

Titration of VEGF with the anti-VEGF FpF antibody mimetics caused an exothermic reaction (Fig. 2) with the derived thermodynamic parameters. The raw heat data and binding isotherm in Fig. 2 showed that the binding interaction between the FpFs and VEGF was an enthalpy driven process, which was probably induced by van der Waals interactions.^14^ A 1 : 1 binding model was applied as the data was best fitted to a one-set-of-sites model.

The high-affinity interaction of the bevacizumab derived antibody mimetic (FpF_beva_) with VEGF was exothermic as shown in Fig. 2. Table 1 summarised the thermodynamic parameters (Δ*H*, −*T*Δ*S* and stoichiometry) for bevacizumab, Fab derived from bevacizumab and FpF_beva_ measured at 25 °C. As with IgGs,^13–15^ the FpF binding was characterised by favourable enthalpy (Δ*H* = −25.7 kcal mole^−1^) and unfavourable entropy (−*T*Δ*S* = 14 kcal mole^−1^) as shown in Table 1. Bevacizumab displayed a Δ*H* = −25 kcal mole^−1^ and −*T*Δ*S* = 13.3 kcal mole^−1^. The binding stoichiometry between FpF_beva_/IgG and homodimer VEGF was measured as a 1 to 1 complex indicating one molecule of FpF/IgG was to bind to one molecule of VEGF.

**Table 1:** ITC thermodynamic parameters for bevacizumab, Fab_beva_ and FpF_beva_. All ITC experiments were carried out at 25 °C and samples were dialysed against PBS pH 7.4 buffer

At 25 °C	Δ*H* (kcal mole^−1^)	−*T*_x_Δ*S* (kcal mole^−1^)	*N*
Bevacizumab	−25	13.3	0.46
Fab_beva_	−12	2.3	0.76
FpF_beva_	−25.7	14	0.55

To compare the differences between thermodynamic binding of bivalent IgG/FpFs and monovalent Fabs, the thermodynamic interaction of free Fab_beva_ with VEGF was evaluated and found to be enthalpy driven and exothermic. Data presented in Table 1 showed the favourable enthalpy (Δ*H* = −12 kcal mole^−1^) and unfavourable entropy (−*T*Δ*S* = 2.3 kcal mole^−1^) of Fab_beva_ were about 2 times for Δ*H* and 5 times for −*T*Δ*S* smaller than thermodynamic parameters reported for full IgG/antibody mimetic.

The stoichiometry of binding between mono-valent Fab and homodimer VEGF was determined as 2 molecules of Fabs needed to bind to 1 molecule of VEGF. Binding stoichiometry of an interaction could imply useful information to confirm protein activity and specificity.^12,26^ The contrasts with the binding thermodynamics of bivalent FpF_beva/_IgG_beva_ compared to the binding thermodynamics of monovalent Fab_beva_ was also shown in the binding interaction of bivalent FpF derived from monovalent Fab_rani_ (ranibizumab) as shown in Table 2.

**Table 2:** ITC thermodynamic parameters for ranibizumab and FpF_rani_. All ITC experiments were carried out at 25 °C and samples were dialysed against PBS pH 7.4 buffer

At 25 °C	Δ*H* (kcal mole^−1^)	−*T*_x_Δ*S* (kcal mole^−1^)	*N*
Ranibizumab	−18.5	6.7	1.09
FpF_rani_	−42	31	0.44

Table 2 shows that while thermodynamic interactions of Fab_rani_ (ranibizumab) with VEGF was exothermic and enthalpy driven with Δ*H* = −18.5 kcal mole^−1^ and −*T*Δ*S* = 6.7 kcal mole^−1^, FpF_rani_ displayed about 2 times larger favourable enthalpy of Δ*H* = −42 kcal mole^−1^ and about 5 times larger unfavourable entropy of −*T*Δ*S* = 31 kcal mole^−1^.

### Binding affinities for IgG and FpFs by ITC

Table 3 lists the comparative affinity values obtained from different techniques used to measure protein–protein interactions. The KD values obtained previously^3^ from SPR and ELISA techniques suggested that bevacizumab (KD_SPR_ = 1.33 nM, KD_ELISA_ = 0.08 nM) and FpF_beva_ (KD_SPR_ = 1.54 nM, KD_ELISA_ = 0.11 nM) displayed similar binding affinity toward VEGF which is consistent to what we obtained from ITC experiments in this work (bevacizumab KD_ITC_ = 2.3 nM, FpF_beva_ KD_ITC_ = 2.1 nM). As expected, monovalent Fab_beva_ displayed lower binding affinity than bivalent antibody in all three techniques.

**Table 3:** KD comparison from ITC, SPR, and ELISA assays

Sample	KD (nM) ITC	KD (nM) SPR	KD (nM) ELISA
Bevacizumab	2.3	1.33	0.08
Fab_beva_	13.9	4.2	0.32
FpF_beva_	2.1	1.54	0.11

### Non-covalent solution binding between FpF and VEGF

**Fig. 3:**
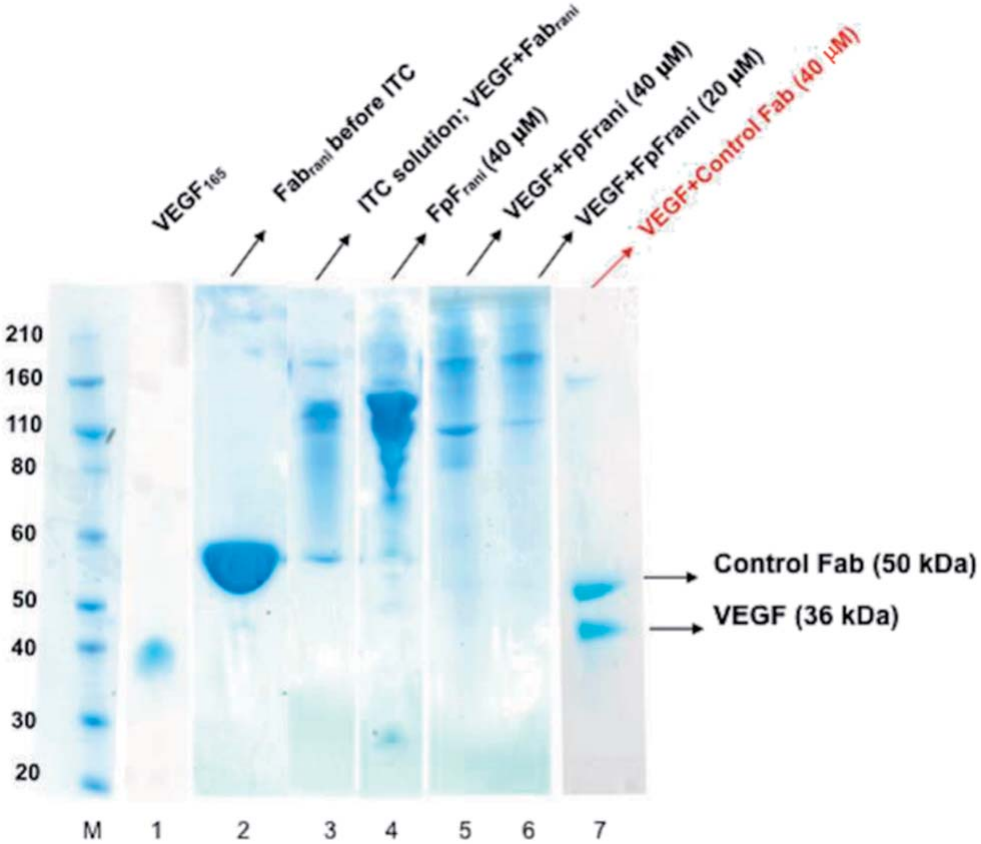
Representative SDS-PAGE for solutions after ITC analysis. Novex bis–tris 4–12% gel stained with colloidal blue for protein. M: standard protein markers, lane 1: VEGF (*M*_w_ = 36 kDa), lane 2: anti-VEGF Fab_rani_, lane 3: ITC solution of reaction between VEGF and Fab_rani_, lane 4: prepared FpF_rani_, lane 5: ITC solution of reaction between VEGF and FpF_rani_ (40 μM), lane 6: ITC solution of reaction between VEGF and FpF_rani_ (20 μM), lane 7: ITC solution of reaction between VEGF and control Fab (40 μM).

The binding interactions between the anti-VEGF antibodies and antibody mimetics with VEGF was visualised by SDS-PAGE. Fig. 3 shows the SDS-PAGE stained with colloidal blue for solutions of VEGF (lane 1), Fab_rani_ before ITC (lane 2), Fab_rani_ after reacting with VEGF (upon completion of the ITC experiment, lane 3), FpF_rani_ before ITC (lane 4), FpF_rani_ after reacting with VEGF (upon completion of the ITC experiment, lanes 5 and 6), and control Fab (non VEGF binder) after ITC analysis (lanes 7). Results (Fig. 3) illustrate the non-covalent binding that occurred when VEGF was titrated with one of the anti-VEGF molecules. For example, as a result of formation of hydrogen bond or van der Waals bond between Fab_rani_ (50 kDa) and VEGF (38 kDa), the band in line 3 was observed at about 90 kDa molecular weight at the saturation phase in ITC experiment. Similar binding interactions were observed for FpF_rani_ with molecular weight of 106 kDa (Fig. 3, lane 4) being titrated with VEGF to show the molecular weight of an approximately 140 kDa (Fig. 3, lanes 5 and 6) at saturation with VEGF. More FpF-VEGF complex was formed when 1 equivalent of VEGF was titrated with FpF (compare lanes 5 and 6). No bond was formed when VEGF was titrated with control, non-VEGF binding Fab as shown in lane 7.

## Discussion

The strength of protein–protein binding is inversely related to the magnitude of the equilibrium dissociation constant, KD. We have previously determined binding affinity (KD) and association, dissociation rate constants (*k*_a_ and *k*_d_) of FpF and analogous Fc-fusion mimetics (RpR) against VEGF using SPR.^3,20^ A RpR is a Fc-fusion mimetic that, in this case combines the VEGF binding moieties of aflibercept through a PEG linking molecule. RpRs address the stability issues that Fc-fusion protein share with IgGs. RpRs displayed greater overall affinity (KD) than Fc fusion. While binding affinity of FpFs was shown to be similar to IgGs, the FpFs displayed slower dissociation rate constants (KD), which would allow FpF to remain in tissue longer than IgGs.

Fabs are linked together in an IgG through relatively short single peptide chains on each heavy chain through the hinge disulfides. In contrast for the FpF, the Fabs are linked through a PEG molecule of 6 kDa. The molecular structures linking the Fabs in an IgG and FpF are very different. Since the Fab CDR structure is the same in both the IgG (bevacizumab) and FpF_beva_, ITC studies are necessary for us to try to understand the impact of the linker molecular structure between the two Fabs. While SPR provides information about the kinetics for binding interactions, ITC provides information about the thermodynamics in solution where these IgG and FpFs function. SPR data can be obscured by mass transfer interactions. Since the Fab linking structures of the IgG and FpF are different, ITC was required to ensure the binding data obtained by SPR was not influenced by mass transfer effects.

ITC is established in life science as ‘gold standard’ and the only method to directly measure binding enthalpy as well as binding stoichiometry. Unlike SPR, ITC determine binding thermodynamic and binding affinity of antibody to the corresponding ligand while both binding partners are in the solution, so no immobilization or chemical modifications are needed. The chemical modification which sometimes are necessary to determine binding affinity in SPR, can potentially interfere or affect binding affinity. In the work described by Bostrom *et al.*^29^ an antibody mimetic against HER2, called bH1, displayed similar binding affinity as trastuzumab (anti-HER-2 antibody) using SPR, but different binding thermodynamics (enthalpy and entropy) using ITC. The interaction of bH1 with the HER2 epitope was entropy driven, whereas binding of trastuzumab to HER2 was enthalpy driven. This information obtained from ITC was important to understand that bH1 has strong structural plasticity resulting in a large favourable entropy change. Such information is helpful to understand the mechanism of interaction to aid the development of new antibody-based medicines.

**Fig. 4:**
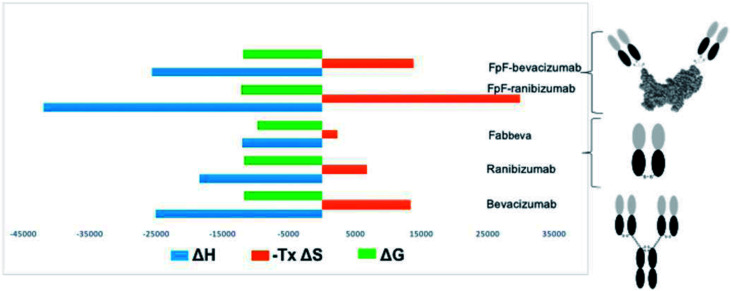
Overview summarising thermodynamic parameters of anti-VEGF molecules. While anti-VEGF antibodies and antibody mimetic (FpF) displayed similar Gibbs energy, the favourable enthalpy observed was a main driving force for forming a complex between anti-VEGF antibody or antibody mimetic and VEGF ligand.

An increase in entropy in protein binding is due to the release of trapped water molecules from proteins into the bulk solvent resulting in more degrees of freedom for water molecule in the bulk compare to the motion restricted water molecules on the protein surfaces.^16,17^ However, release of water molecules from a protein at the binding interface may also result in structural rearrangements such as the closure of hydrophilic cavities and release of bound water from the binding interface.^17^ Binding of bevacizumab and FpF_beva_ to VEGF is driven by enthalpy which is greater than the entropic cost (Fig. 4). While they displayed similar Gibbs energy, the favourable enthalpy observed was a main driving force for forming a complex between anti-VEGF antibody or antibody mimetic and VEGF ligand.

Unfavourable entropy is thought to be a result of conformational change at binding interface leading to a lower degree of freedom for the formed complex. For example, Seroussi *et al.* used ITC to investigate the structure activity relationships of different anti-VEGF cyclic peptides. Results from alanine mutation demonstrated that the binding affinity was lost for the isomer with the lowest positive entropy change.^28^ Larger entropic compensation and less degree of movement was observed in the anti-VEGF FpF_rani_ binding to VEGF compared to ranibizumab suggesting that when one Fab in FpF is bound to VEGF dimer, the other Fab would bind to the same VEGF dimer while ranibizumab as a monovalent Fab has more degrees of movement for binding to a different VEGF ligand. This result suggested that FpF_rani_ comprising of two ranibizumab Fabs would tightly bind to VEGF. Consideration that dissociation rate of ranibizumab is exceptionally slow,^18^ an FpF derived from ranibizumab would be an exceptionally tight binding molecule.

Data obtained from SPR,^3^ suggests there is similar binding affinity but slower dissociation rate constant for antibody mimetic (FpF_beva_, FpF_rani_) compare to the parent native antibody (bevacizumab, ranibizumab). Slower dissociation rates are therapeutically important to ensure that once bound to a ligand, an antibody does not in effect become a slow releasing depot for that ligand. It also offers a viable strategy to increase efficacy by increasing the residence time within the target tissue.^19^ One reason that ranibizumab has been such a successful drug is because its dissociation rate is exceptionally slow, can only be measured with difficulty.^19^ Hence, slower dissociation rate and larger unfavourable entropy could suggest a potential for development of longer lasting antibody mimetics which require less frequent of dosing.

In order to design a new therapeutic drug and engineer new antibody mimetics, more complete understanding of the interactions between antibody and its target epitope are necessary. Insight leading to better therapeutic design does not simply occur by knowing the binding affinity. A full thermodynamic analysis to determine enthalpy, entropy, binding stoichiometry of interaction, are required to provide the information related to a molecular force at binding interface between antibody and the ligand.

## Experimental

### Materials

Bevacizumab (Avastin®, 25 mg mL^−1^, Genentech) and ranibizumab (Lucentis®, 10 mg mL^−1^, Genentech) were obtained as clinical leftover. hVEGF_165_ was purchased from Peprotech. Slide-A-Layzer dialysis cassette kit, 3.5 K MWCO, 0.5 mL was purchased from ThermoFisher scientific. Phosphate buffered saline (PBS; 0.16 M NaCl, 0.003 M KCl, 0.008 M Na_2_HPO_4_ and 0.001 M KH_2_PO_4_) was prepared with tablets purchased from Oxoid.

### Methods

Fab_beva_ was obtained by the enzymatic digestion of bevacizumab using immobilized papain as described previously.^3^ Purified Fab_beva_ was isolated from the digestion mixture using a Protein A column and then buffer-exchange to PBS buffer using Slide-A-Layzer dialysis cassette with 3.5k cut off overnight at 4 °C.

Fab_beva_ (2.4 mg mL^−1^, 6.0 mg in 2.5 mL PBS, pH 7.3) was incubated with dithiothreitol (DTT) (1.0 mg mL^−1^, 2.5 mg) at ambient temperature without shaking for 30 min. DTT was then removed by elution over a new PD-10 column, and the protein was buffer exchanged into the conjugation buffer (20 mM sodium phosphate, 10 mM EDTA, pH 7.4). PEG-di(bis-sulfone) 1 (0.9 eq, 6 kDa) was added (1.08 mg) to the reduced Fab_beva_ solution (6.0 mg in 3.3 mL). The solution was incubated at ambient temperature for approximately 3 h without shaking. FpF_beva_ was purified using a single step HiTrap Macrocap SP cation exchange column (IEC-Macrocap SP, 5.0 mL). The concentration of the purified FpF_beva_ was calculated by micro BCA assay using bevacizumab as standard. Similar procedures were applied to prepare FpF_rani_ using Fab_rani_ for conjugation with PEG-di(bis-sulfone) 1.

ITC measurements were performed in a MicroCal™ iTC_200_ with 200 μL sample cell at 25 °C. Extra care was taken when preparing the samples for ITC experiments as impurities can significantly affect the experiment. All ITC samples including VEGF_165_ (4 μM, 250 μL), and the titrants (20 μM bevacizumab, 40 μM Fab_beva_, 20 μM FpF_beva_, 40 μM Fab_rani_ and 20 μM FpF_rani_) had to dialyzed against PBS (pH 7.4) using Slide-A-Layzer dialysis cassette with 3.5k cut off for overnight at 4 °C. The titrants were placed in the syringe, and 2 μL aliquots were injected incrementally into the sample cell containing VEGF_165_ (4 μM, 250 μL) at duration of 4 s, spacing of 200 s while stirring at 1000 rpm. Titration of PBS to PBS and VEGF to PBS were used as a control. The correction was applied on the baseline for the heat of dilution in the sample due to addition of the sample by subtracting the integrated peak area from an estimated constant number which was derived from the plot between the molar ration of titrant *versus* titrand. The binding stoichiometry (*N*), affinity (KD), binding constant (*K*_B_) and enthalpy change (Δ*H*) were determined by fitting the data using the “one-set-of-sites” independent binding model^21–23^ provided by MicroCal software. KD = 1/*K*_B_; Δ*G* = *RT* ln KD, where *R* is the gas constant (8.315 J K^−1^ mol^−1^) and *T* is the absolute temperature. Δ*S* values were obtained by calculation using the equation *T*Δ*S* = Δ*H* − Δ*G*.

## Conclusions

Using ITC enabled us to study the mechanism of interaction between anti-VEGF antibody mimetics (FpF) synthesised from bevacizumab and ranibizumab to circulating VEGF. In conclusion, while anti-VEGF antibody mimetic FpF_beva_ displayed similar binding affinity (KD) and binding thermodynamics (Δ*H*, Δ*S*) as bevacizumab, FpF_rani_ showed to have larger favourable enthalpy and unfavourable entropy than ranibizumab.

The results suggest that the antibody mimetics, are highly flexible and undergoes a conformational change combined with significant interface de-solvation upon binding to VEGF. These physicochemical properties of FpF enable the requirements for antibody binding which is a primary factor underlying its antigenic potential.

Antibody mimetics such as FpFs have potential for next generation therapies because of their enhanced protein stability, the lack of Fc effector function, and high binding affinity and binding thermodynamics with large unfavourable entropy. In particular there is potential to develop FpFs for intraocular use to treat blinding conditions such as wet age macular degeneration (AMD) eye.

## Conflicts of interest

The authors declare no competing financial interest.

## Supplementary Material
